# 30-yr course and favorable outcome of alveolar echinococcosis despite multiple metastatic organ involvement in a non-immune suppressed patient

**DOI:** 10.1186/1476-0711-12-1

**Published:** 2013-01-02

**Authors:** Karine Bardonnet, Dominique A Vuitton, Frédéric Grenouillet, Georges A Mantion, Eric Delabrousse, Oleg Blagosklonov, Jean-Philippe Miguet, Solange Bresson-Hadni

**Affiliations:** 1 WHO Collaborating Centre for Prevention and Treatment of Human Echinococcosis, University Hospital, University of Franche-Comté, Besançon 25030, France; 2 Department of Biochemistry, University Hospital, Besançon 25030, France; 3 EA 3181 “Epithelial carcinogenesis: prognosis and prediction factors”, University Hospital and CNRS, University of Franche-Comté, Besançon 25030, France; 4 Dept of Parasitology, University Hospital, Besançon 25030, France; 5 UMR 6249 Chrono-Environment Joint Research Unit, University Hospital and CNRS-INRA, University of Franche-Comté, Besançon 25030, France; 6 Deparment of Digestive Surgery, University Hospital, Besançon, 25030, France; 7 Deparment of Radiology, University Hospital, Besançon, 25030, France; 8 Department of Nuclear Medicine, University Hospital, Besançon, 25030, France; 9 Department of Hepatology, University Hospital, Besançon, 25030, France

## Abstract

We report the 30-yr history of a well-documented human case of alveolar echinococcosis, with a lung lesion at presentation followed by the discovery of a liver lesion, both removed by surgery. Subsequently, within the 13 years following diagnosis, metastases were disclosed in eye, brain and skull, as well as additional lung lesions. This patient had no immune suppression, and did not have the genetic background known to predispose to severe alveolar echinococcosis; it may thus be hypothesized that iterative multi-organ involvement was mostly due to the poor adherence to benzimidazole treatment for the first decade after diagnosis. Conversely, after a new alveolar echinococcosis recurrence was found in the right lung in 1994, the patient accepted to take albendazole continuously at the right dosage. After serology became negative and a fluoro-deoxy-glucose-Positron Emission Tomography performed in 2005 showed a total regression of the lesions in all organs, albendazole treatment could be definitively withdrawn. In 2011, the fluoro-deoxy-glucose-Positron Emission Tomography showed a total absence of parasitic metabolic activity and the patient had no clinical symptoms related to alveolar echinococcosis.

The history of this patient suggests that multi-organ involvement and alveolar echinococcosis recurrence over time may occur in non-immune suppressed patients despite an apparently “radical” surgery. Metastatic dissemination might be favored by a poor adherence to chemotherapy. Combined surgery and continuous administration of albendazole at high dosage may allow alveolar echinococcosis patients to survive more than 30 years after diagnosis despite multi-organ involvement.

## Introduction

Alveolar Echinococcosis (AE) caused by the metacestode of the “fox tapeworm” *Echinococcus (E.) multilocularis* is one of the most lethal helminthic diseases in humans [1,2]. Humans become infected through contact with eggs (oncospheres) present in the feces of the definitive hosts, most often foxes or dogs, but also wolves and cats, by handling the animals or by ingesting contaminated vegetables without cooking them. Only observed in the northern hemisphere, and especially in central Europe, Russia/Siberia, Central Asia, Western China, north of Japan, and Alaska, AE is also one among the rare parasitic diseases with a surgical treatment, because of its “tumor-like” progression. The only available antiparasitic chemotherapy, *i.e*. high doses of albendazole (ABZ) or mebendazole (MBZ) given continuously, is only parasitostatic in this disease and the complete resection of the parasitic lesions is thus recommended whenever possible [3,4]. In most of the cases, the parasitic lesions are initially located in the liver and may then invade adjacent organs; however, true metastases may also be seen in any organ or tissue. Extra-hepatic locations were already present at diagnosis in 34% of cases in those cases recorded in the EurEchinoReg European registry from 1982 to 2000 [5]. When the clinical symptoms at presentation are related to such extra-hepatic locations, the diagnosis of AE is difficult and very often it is confirmed after surgery on the pathological aspects of the lesions and/or evidence of its parasitic nature by PCR [6,7]. Except for lung metastases, AE metastatic dissemination is often associated with immune deficiency of the host [8]. “Multi-organ AE” usually qualifies cases with hepatic, pulmonary and cerebral locations [9,10]; metastases to more than 2 organs/tissues are extremely rare, either simultaneously or successively, in immune-competent subjects. The involvement of several organs is among the main causes of poor prognosis [11,12]. We report here the case of an immune-competent man with an AE discovered from a lung metastasis in 1981 who had 5 different locations of the disease, either simultaneously or successively, underwent 4 surgical operations on 6 different organs/tissues in 4 different locations, and who may nevertheless be considered cured from the disease after a unique follow-up of more than 30 years.

## Case report

**Figure 1:**
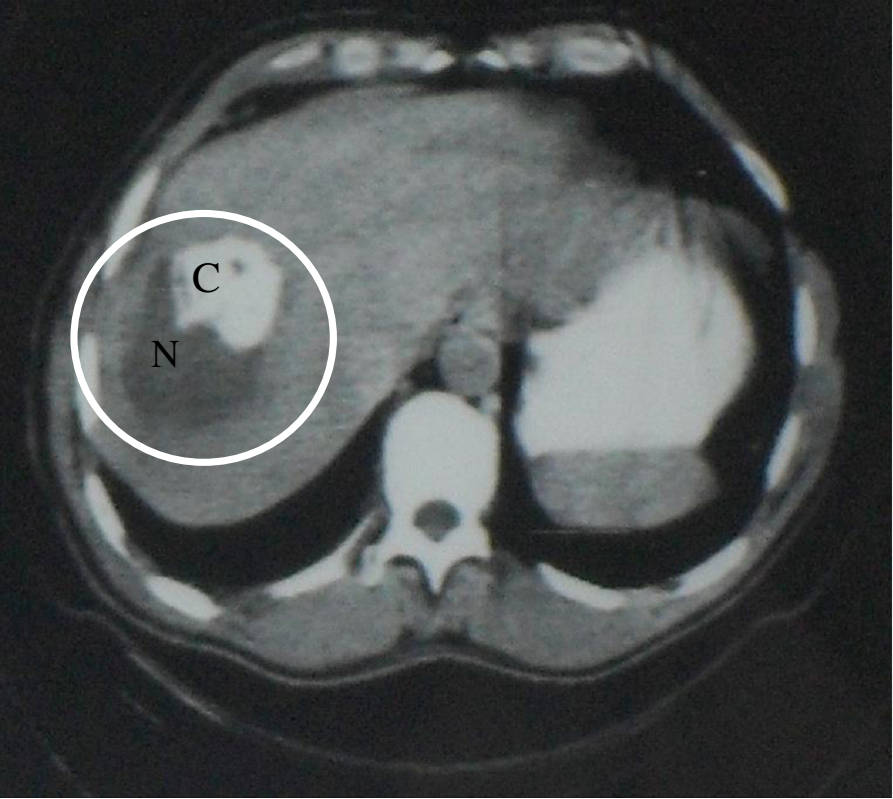
Abdominal CT Scan (1982): alveolar echinococcosis heterogeneous lesion of the right lobe of the liver, including a large necrotic area (N) and a calcified area (C).

The patient was a 38-year-old man who was admitted into a local hospital of the region of Franche-Comté, Eastern France, for the cure of an inguinal hernia. Systematic surgery pre-assessment disclosed by chance a lung nodule which evoked pulmonary cancer. Thoracotomy with right upper lobectomy of the lung was performed in January 1982 in Dijon University Hospital, France, and the fortuitously discovered “tumor” was totally removed. At the end of the operation, through the diaphragm, the liver appeared to be enlarged, with a modified structure, and the surgeon found a liver tumor with a very hard consistency and a necrotic content of 100 mL which was aspirated; the diagnosis of AE was thus evoked. Pathological examination of the lung “pseudo-tumor” and of the surgical biopsies taken after diaphragm incision, as well as the Computed Tomography (CT) images obtained after the operation (Figure 1), confirmed the diagnosis. As the patient wished to postpone the proposed liver resection, flubendazole was given to the patient at a daily dosage of 9.0 g [13] until 1985 when he was eventually transferred to Besançon University Hospital for radical liver resection and inclusion in chemotherapy trials. The patient gave his consent to be included in clinical research studies within the framework of the study “Immunogenetics of AE in humans” first, in 1994, then as part of the prospective follow-up of AE patients in the FrancEchino Registry in 2003; both prospective cohort studies were approved by the French legal ethical committee (CCPPRB/Franche-Comté: comité consultatif pour la protection des personnes en recherche biomédicale, for the region of Franche-Comté); written informed consent was obtained from the patient for publication of this report and any accompanying images.

In May 1985, the specific serology tested by ELISA using crude larval Em (EmC), and purified Em2 as antigens [14] was positive but in favor of a stable disease, as well as the unchanged CT images which showed the well-limited parasitic mass in the right liver with partial necrosis and calcifications, and no involvement of the biliary tree and/or hepatic and portal vessels. A right hepatectomy with cholecystectomy was thus performed, which removed a 1.45 kg parasitic tumor and was considered to be curative. Flubendazole was replaced by MBZ at a daily dosage of 4.5 g from June to December 1985; then replaced by ABZ at a daily dosage of 400 mg twice a day for 1 month followed by 2 week-interruptions of treatment as recommended by the manufacturer at that time (‘cyclic administration’) [3]. MBZ and ABZ were given within the framework of WHO-coordinated therapeutic trials [15,16] and the patient had a regular prospective follow-up in Besançon University Hospital WHO-clinical reference Centre. ABZ treatment was spontaneously interrupted by the patient himself in June 1986, because of minor subjective side-effects (digestive discomfort) without any biological abnormalities. The previous treatment by MBZ was then resumed. In May 1988, there were no clinical or biological abnormalities; ultra-sound examination only showed the expected enlargement of the left liver usually observed after right hepatectomy and the patient was considered to be cured. As serology (ELISA using crude larval antigen EmC, and purified Em2) was fully negative and liver and lung imaging remained normal, MBZ was thus stopped on May, 1988.

In April 1991, the patient was admitted into the University Hospital/AE reference Centre with a 3-month long history of headache and right exophtalmia; visual acuity, ocular mobility and eye fundus examinations were normal. A re-increase in specific antibodies in the serum was disclosed although abdominal and thoracic imaging showed no recurrence of AE in the liver or adjacent organs, or in the lung. Cerebral CT-scan and Magnetic Resonance Imaging (MRI) disclosed 2 lesions: one in the orbit and one in the right frontal lobe of the brain. Both had a micro-polycystic hypodense structure, with scattered calcifications. The surgical treatment consisted of a subtotal resection of the lesion in the right frontal lobe, the adjacent dura, the invaded part of the eye lid and the eroded frontal bone. Brain and right orbital AE, with bone involvement was confirmed by the pathological examination of the lesions which showed necrotic and fibrous tissue filled with small cysts. Microscopically, typical *E multilocularis* germinal layer and protoscoleces were observed as well as periparasitic epithelioid cells and the granulomatous infiltrate of macrophages, lymphocytes and giant cells. ABZ at a daily dosage of 800 mg, following the “cyclic” administration as before, was introduced again in June 1991, with recommendations of strict adherence to the treatment. At the end of 1991, the situation was clinically stable, with a marked regression of the remaining orbital and cerebral lesions. Once again, upon the patient’s demand, because of digestive discomfort, in March 1992, ABZ was switched to MBZ; then the patient spontaneously interrupted his treatment in July 1992. ABZ was resumed in October 1992 when the patient complained of ptosis of the right eye and reduction of his visual acuity, despite unchanged images of the brain and orbital region. But once again the patient stopped his treatment in February 1993.

**Figure 2:**
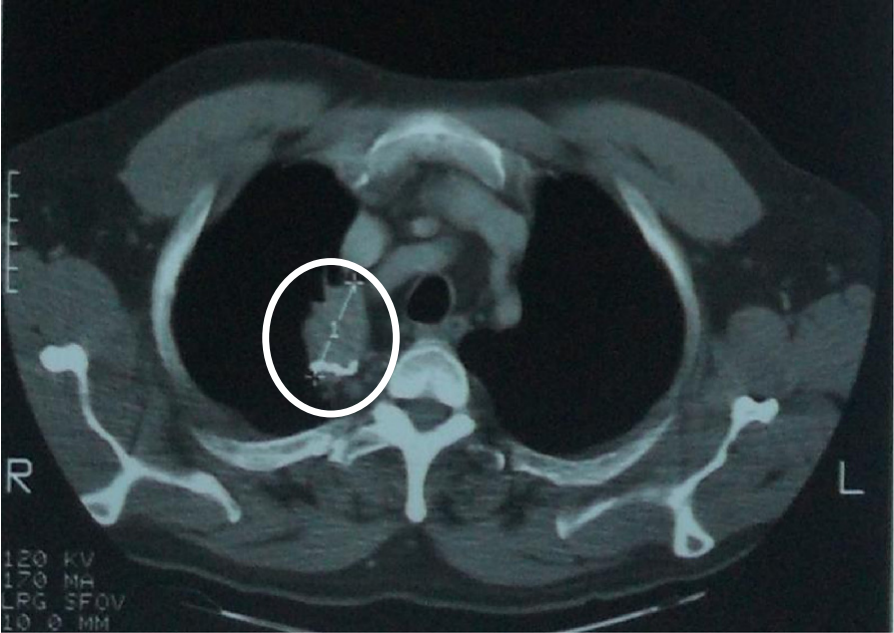
Thoracic CT Scan (1994): recurrent pulmonary lesion of alveolar echinococcosis after the right lobectomy performed in 1982.

**Figure 3:**
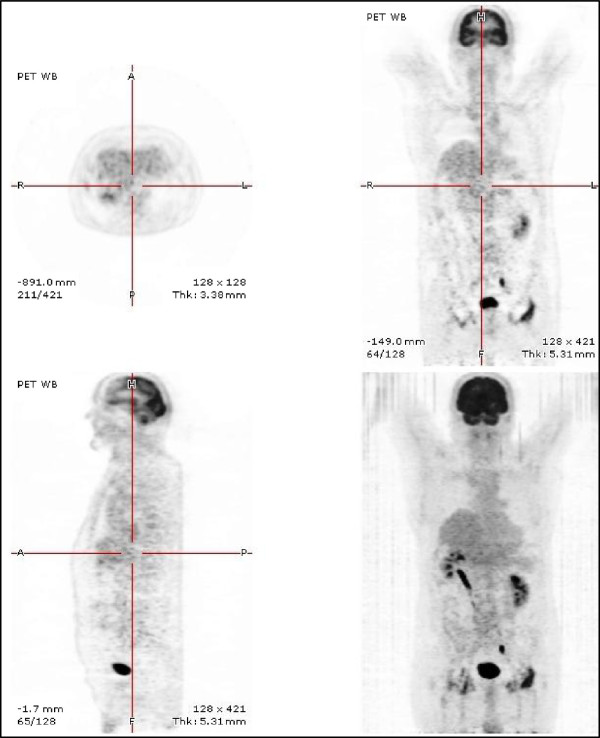
Positron Emission Tomography combined with CT Scan (2011): no FluoroDeoxyGlucose uptake whatever the previously involved organ.

At the beginning of 1994, thoracic CT-scan showed AE recurrence, as a 45 mm in diameter-lesion in the right pulmonary apex (Figure 2). From that date, the patient accepted to take ABZ at an increased dosage (20 mg/day/kg) and continuously, as suggested by the recommendations of the WHO-Informal Working Group on Echinococcosis for severe AE cases (1996 Guidelines). In February 1996, as AE seemed under control, the 52-yr old patient benefited from an elective left hip prosthesis in that same local hospital he had been admitted first in 1981. Because of an abnormal structure of the bone disclosed by the surgeon, a pathological examination was performed in Besançon University Hospital on bone sections. Despite abnormalities compatible with parasitic vesicles, formal AE diagnosis could not be ascertained since no typical germinal layer was observed; the inflammatory infiltrate could also be due to associated arthritis; and no PCR could be performed retrospectively on the lesions because of technical issues. Complete clinical and radiological assessment of possible metastatic locations of the disease did not disclose any other lesions. In 1999, the size of the pulmonary lesion had markedly decreased (to 21 mm in diameter) and in 2000 serology (Em2 and EmC ELISA) was completely negative. Since then, the patient has been in good health, except for overweight (height: 178 cm; weight: 100 kg; abdominal perimeter: 117 cm) and subjective complaints such as headaches and pain in his left hip. Adherence to ABZ treatment was good. Yearly imaging exams including ultrasound examination and CT-scans did not show any changes in the images. A Fluoro-DeoxyGlucose (FDG)-Positron Emission Tomography (PET) combined with Computed Tomography (CT) performed in 2003 showed the absence of FDG uptake by the lesions in all organs, 1 h after FDG injection. Serology (Em2 and EmC ELISA) remained negative and ABZ was thus withdrawn definitively in April 2003. At last follow-up in 2011, there was no evidence of recurrence/relapse, as evidenced by negative PET-CT with conventional and delayed acquisition of the images 3 h after FDG injection (Figure 3), and by negative Em2+ ELISA serology (Bordier Affinity Products, Crissier, Switzerland) [17]. At that time, serology was also assessed by *E. multilocularis* western-blot (LD Bio, Lyon, France) [18] in parallel on two sera: a sample frozen in 1987, and a freshly collected sample. With the serum sampled in 1987, two bands at 7 kDa and 26-28 kDa were observed, while with the serum collected in 2011 only binding to 26-28 kDa *Echinococcus* antigens was observed. Disappearance of the 7 kDa band thus confirmed imaging and ELISA serology results.

## Discussion

In the 1970s, in the patients with AE, life expectancy was estimated to be reduced by 18.2 and 21.3 years for men and women respectively; by 2005 it was reduced by approximately 3.5 and 2.6 years, respectively [19]. Continuous ABZ has greatly contributed to prolonged survival [12,20]. Presence of metastasis is generally considered of poor prognosis in AE; a recent study on 387 French patients confirmed that it was actually associated with higher AE-specific mortality [12]. Moreover, multiple extra-hepatic locations of AE are usually associated with immune suppression [8]. The history of the AE patient we are reporting suggests that multi-organ involvement and AE recurrence over time may occur in non-immune suppressed patients despite an apparently “radical surgery” which removed all visible AE lesions, and might be favored by a poor adherence to the benzimidazole chemotherapy. It also shows, however, that combined surgery and continuous administration of ABZ at high dosage may allow patients to survive more than 30 years after diagnosis despite multi-organ involvement.

Occurrence of metastatic locations of AE lesions is one of the hallmarks which justify the similarities between AE and malignant tumors, as exemplified by the PNM classification which parallels the TNM classification of tumors [21]. Invasion of neighboring organs and tissues (“N” in the PNM classification) by *E. multilocularis* progression from the initial liver location results in secondary lesions in the right lung through the diaphragm, in peritoneal and retroperitoneal cavities, hepatic pedicle, round ligament, right kidney and adrenal gland, pancreas, stomach or spleen. True metastases (“M” in the PNM classification) are mainly observed in the lung (7% of cases) [5], more rarely brain (3%), spleen (1%) [5] or bones (less than 1%) [6,22], in the skin [23] muscle [24], heart [24,25], and in any possible anatomical locations [2]. The clinical presentation of our patient combined all main 3 locations; the brain location was associated with orbit, eye, and facial/cranial bone involvement, which is *per se* very rare and was for that reason published several years ago [26]. In addition, metastases occurred along a 13-year period, from the patient’s inaugural presentation with a lung metastasis in 1981 to the last discovery of another lung metastasis in 1993; meanwhile, brain, eye and bone locations were disclosed. In otherwise non-immune suppressed patients, genetic factors leading to poor cellular immunity and a marked trend to immune tolerance are statistically associated with the severity of AE, which includes metastasis formation [27,28], *e.g.* HLA B8, DR3, DQ2 haplotype; however, this ‘at risk’ haplotype was not present in our patient which actually was HLA A11, A10; B13, B41; DR7, DR13; DQ2, DQ3. The main risk factor for disseminated AE is actually immune suppression. First observations were reported after liver transplantation for AE in patients who were not treated by benzimidazoles after receiving the liver graft [29]. Increase in residual extra-hepatic lesions as well as occurrence of brain or spleen metastasis were observed in a European series of 45 transplanted patients [30]. In the 1990s, such observations contributed to greatly reduce the indication of liver transplantation to treat AE [3]. Since then, AE occurrence in patients with kidney or heart transplantation has also been reported [31,32]. Rapidly progressing AE was also observed in AIDS [33,34] and it was suggested that pregnancy could be a tolerogenic situation which may have favored occurrence of brain metastasis [35]. Within the recent years, development of cancer chemotherapy and use of more potent immune suppressive drugs as well as immune modulating biological agents, especially anti-TNF compounds, have contributed to an increase in the number of disseminated and/or rapidly progressing “opportunistic AE” in patients with cancer, hematological disorders or chronic inflammatory diseases [7,36,37]. Our patient, however, all along his clinical course, did not present any overt - spontaneous or disease/treatment-related- immune suppression. There is no reason either to think that he was infected by an unusually virulent strain of *E. multilocularis:* several patients were diagnosed with slowly progressing, non-disseminated AE within a 30 km-range from his residence; and recent genetic analyses of *E. multilocularis* in the patient’s endemic area do not favor major differences which might be responsible for more or less aggressive potential of the various strains [38]. Absence of the highly specific *E. multilocularis* 16-18 kDa band at Western Blot on the serum sampled in 1987, a few months before the first treatment withdrawal, was compatible with the negative ELISA results at that time, and thus with “inactive” lesions; however, “inactivity” was only temporary, as the following evolution well demonstrated.

First introduced in the pharmacopeia at the end of 1970s, benzimidazoles, although they do not kill *E. multilocularis* larvae, are the only drug available to treat AE and, being able to prevent metacestode growth and provided they are taken for life, have markedly contributed to improve AE patients’ prognosis [11,12,19]. Albendazole, taken at the appropriate dosage of at least 15 mg/kg/day according a continuous schedule, is currently considered as the drug of choice [4]. Bad adherence to treatment, initial treatment with flubendazole which was later proved inefficient in AE, iterative switches from ABZ to MBZ because of side-effects, as well as insufficient dosage of ABZ due to the “discontinuous” schedule of administration recommended during the 1980-1990s [3] may have been the main reasons for repeated dissemination of AE lesions in the reported patient’s. Adherence to treatment is essential and bad adherence, or ABZ withdrawal because of side-effects, have been shown to be crucial for lesion recurrence in patients with residual lesions after liver transplantation [20]. It is remarkable that, after 13 years of unreliable intake of the antiparasitic drugs tainted with successive extra-hepatic metastases of the disease, good adherence to ABZ treatment was followed by the inactivation of the lesions, negative PET-CT images and serology within 10 years, and a decision of definitive treatment withdrawal. The relative ‘tolerance’ of physicians in charge of the patient towards his bad adherence to treatment may also have been encouraged by the overconfidence of the surgeons about the complete resection of the initial lung and liver lesions. In addition, the absence of any reliability in the patient’s allegations regarding last intake of the antiparasitic drug prevented them to measure ABZ sulfoxide plasma levels, which is, however, usually routinely performed in that reference center; MBZ measurements were not available when the patient switched to MBZ, which he did most of the time; and lower bioavailability of MBZ is well known [3]. Difficulties to assess completeness of the surgical resection and necessity to adjunct benzimidazoles even if resection was considered to be curative, were stressed as early as the 1980s [14,39] and were further highlighted by the observations of recurrence after liver transplantation [30]. Measurement of ABZ levels and proper interpretation of low levels of ABZ sulfoxide as evidence of bad adherence to treatment are also essential in the follow-up of AE patients [4].

It is fortunate that our patient is still alive 30 years after the diagnosis of AE and 9 years after antiparasitic drug withdrawal; this observation confirms the therapeutic effectiveness of ABZ, despite its absence of parasitocidal effect, and possible withdrawal of the drug in highly selected subjects. Absence of FDG uptake at PET-CT assessment and negative Em2+ ELISA serology in 2011 suggest an aborting evolution of the parasitic lesions whatever the involved organ or tissue; persistence of the 26-28 kDa band at Western Blot is likely related to the persistence of non-viable parasitic tissue in various organs of the patient, as is often the case in AE patients with aborted lesions [2]. The current recommendations concern the “security margin” to be observed by the surgeons for the resection of AE lesions, similar to those recommended in oncological surgery, as well as the 2-yr ABZ continuous treatment which is mandatory in the so-called “radically operated on” patients [4]. Following these recommendations would have likely prevented the occurrence of the distant metastases and also radically changed our patient’s quality of life.

## Competing interest

The authors who have taken part in this study declare that they do not have anything to disclose regarding funding or any other conflict of interest with respect to this manuscript.

## Authors’ contributions

KB, DAV and SBH were the primary authors for the manuscript. DAV and SBH participated in drafting the manuscript. FG assisted in data gathering and helped in drafting the manuscript. GAM, ED, OB, JPM assisted in data gathering and participated in the design of the case report. All authors made substantial contributions to the acquisition of data. All authors read and approved the final manuscript prior to publication.
